# Development of a Multilayer Film Including the Soluble Eggshell Membrane Fraction for the Treatment of Oral Mucosa Lesions

**DOI:** 10.3390/pharmaceutics16101342

**Published:** 2024-10-19

**Authors:** Karthik Neduri, Giorgia Ailuno, Guendalina Zuccari, Anna Maria Bassi, Stefania Vernazza, Anna Maria Schito, Gabriele Caviglioli, Sara Baldassari

**Affiliations:** 1 Department of Pharmacy, University of Genova, 16148 Genova, Italy; karthikneduri@gmail.com (K.N.); giorgia.ailuno@unige.it (G.A.); guendalina.zuccari@unige.it (G.Z.); gabriele.caviglioli@unige.it (G.C.); 2 Department of Experimental Medicine, University of Genova, 16132 Genova, Italy; anna.maria.bassi@unige.it (A.M.B.); stefania.vernazza@unige.it (S.V.); 3 Inter-University Center for the Promotion of the 3Rs Principles in Teaching & Research (Centro 3R), 16132 Genova, Italy; 4 Department of Surgical Sciences and Integrated Diagnostics, University of Genova, 16132 Genova, Italy; amschito@unige.it; 5 IRCCS Ospedale Policlinico San Martino, 16132 Genova, Italy

**Keywords:** mucoadhesion, buccal films, solubilized eggshell membrane, chlorhexidine digluconate, cancer oral lesions

## Abstract

Background/Objectives: Oral diseases causing mucosal lesions are normally treated with local or systemic anti-inflammatory, analgesic and antimicrobial agents. The development of topical formulations, including wound-healing promoters, might speed up the recovery process, improving patients’ quality of life, and reduce the risk of deterioration in health conditions. In this study, a mucoadhesive multilayer film, including a novel biocompatible substance (solubilized eggshell membrane, SESM), was rationally designed. Methods: The SESM preparation procedure was optimized and its biological effects on cell proliferation and inflammation marker gene expression were evaluated in vitro; preformulation studies were conducted to identify the most promising polymers with film-forming properties; then, trilayer films, consisting of an outer layer including chlorhexidine digluconate as a model drug, a supporting layer and a mucoadhesive layer, incorporating SESM, were prepared using the casting method and their mechanical, adhesion and drug release control properties were evaluated. Results: SESM proved to possess a notable wound-healing capacity, inducing a wound closure of 84% in 24 h without inhibiting blood clotting. The films revealed a maximum detachment force from porcine mucosa of approx. 1.7 kPa and maximum in vivo residence time of approx. 200–240 min; finally, they released up to 98% of the loaded drug within 4 h. Conclusions: The formulated trilayer films were found to possess adequate properties, making them potentially suitable for protecting oral lesions and favoring their rapid healing, while releasing antimicrobial substances that might be beneficial in reducing the risk of bacterial infections.

## 1. Introduction

The Global Oral Health Status Report 2022 estimated that oral diseases affected 3.5 billion people worldwide in 2019, comprising the latest data available [1]. Some of them, such as cancer-related mucositis, periodontitis and aphthous ulcers, cause lesions of the mucosa, which are painful and difficult to treat.

Radiation-induced oral mucositis (RIOM), a tissue injury caused by radiotherapy, occurs in up to 80% of head and neck cancer irradiated patients [2], leading to marked adverse effects on patient’s quality of life, since it is usually associated with intense pain, increased opioids consumption, increased risk of bacteremia [3] and often cancer therapy interruption [4]. The treatment of RIOM is symptom-based [5].

Periodontitis, with approximately 1 billion cases [6], is a chronic disease comprising various degenerative and inflammatory states in the gums, periodontal ligaments and alveolar bone [7], with the proliferation of pathogenic microbes and, in the most advanced cases, tooth dislodgement.

Oral aphthous ulcers are the most common oral mucosal lesions, with a wide range of reported prevalence (from 5 to 25%) in different populations; they can cause severe pain, reducing the patients’ quality of life. Also in this case, as their etiology is unknown, their treatment is symptom-based [8].

Oral lesions are normally treated aspecifically with local or systemic anti-inflammatory agents, analgesics and antimicrobials; among them, chlorhexidine (CHX) salts, especially in the form of mouth rinses, are frequently used to relieve the symptoms and reduce their duration. CHX exerts its antimicrobial activity through its positive charge at a physiological pH, which destabilizes bacterial cell walls and alters bacterial osmotic equilibrium, triggering microbial cell death [9]. However, CHX, although it reduces the bacterial load in the oral cavity and prevents concomitant infections, should not come into contact with mucosal lesions, as several experimental studies suggest that it may interfere with the healing process [9,10,11,12,13,14,15,16,17,18,19,20,21].

Independently from their primary cause, in all oral conditions causing the formation of lesions, a local formulation including a material that speeds up wound healing should be used, and, in pursuit of this aim, many natural polymers, like dextrans, chitosan, alginate and fucoidan, have proven to be suitable for wound dressing, while exerting a useful co-adjuvant antibacterial activity [22,23,24,25]. Moreover, to reduce the overall bacterial load, CHX could be associated with these formulations, but would ideally be incorporated into a system able to control its release rate in the oral cavity, while limiting its direct release into the lesion.

Among the feasible formulations for this application, multilayer films have shown significant advantages over polymeric single-layered films or monolithic films, such as increased drug loading and the possibility to segregate incompatible components [26,27,28]. They are also preferred over adhesive tablets because they are flexible and comfortable to use [29] and enable the inclusion of actives whose release should occur in different directions [30,31], either towards the mucosa below them or towards the oral cavity, depending on the mechanism of action and the pathology to be treated.

An efficacious polymeric film should be bioadhesive, resistant, biocompatible with the unhealthy tissue and able to release the loaded drug at a controlled rate [32,33,34]. The adhesion properties of a film depend on its composition, and, in principle, can be adjusted by carefully selecting its polymeric ingredients, so that it can adhere to the mucosa without causing damages upon removal. Moreover, an ideal film should be elastic, flexible and strong enough to withstand the stress it undergoes during its residence in the mouth.

Hence, in the present study, the rational design of a mucus membrane-adhering multilayer film is described. The aim of this work was to develop a formulation to be applied on buccal lesions, ulcers and wounds, independently from their origin, which was able to protect them from the pathogens present in the saliva and promote their healing. This multilayer film was composed of a mucoadhesive layer, a supporting layer and a backing layer, and was conceived to be used as a promising platform for wound-healing applications in oral diseases.

For this purpose, a preliminary preformulative study on different film-forming polymers was conducted, aimed at selecting the best candidates for the future development of a multilayer film. Then, the film manufacturing process, based on the casting procedure, was optimized.

The mucoadhesive layer of an ideal multilayer film must have a barrier function, thereby protecting the wound or injury from the oral environment, and should promote the healing process. For this reason, a mucoadhesive polymer (hydroxypropyl cellulose, HPC) was combined with a substance of animal origin, namely the solubilized fraction of eggshell membrane (SESM), which is a biocompatible material that contains, among other minor components, hyaluronic acid, collagen, elastin and chondroitin-4-sulphate, biopolymers that might favor the repair of the lesion [35,36,37,38]. Actually, for more than four hundred years, eggshell membrane (ESM) has been used to cure injuries, and is mentioned in the pharmacopoeia of Chinese medicine, Bencao Gangmu [39]; many formulations, including a medical device recently patented and placed on the market (Dermarep^®^), have been prepared using ESM particulate, which, however, also includes non-bioavailable insoluble components [40,41]. Hence, converting the ESM components into smaller, water-soluble molecules might increase their wound-healing potential.

SESM can be obtained through the acidic treatment of ESM [41] or through alkaline hydrolysis [39,42]. For its application in the multilayer films reported hereafter, a previously described alkaline procedure was optimized [35], and the final product was evaluated for cytocompatibility and its anti-inflammatory and wound-healing properties.

The backing layer of the multilayer film was conceived to control the release of drugs useful for the prevention of bacterial infection of the wound site, while limiting the direct release of the active drug within the lesion, as it might compete with the healing activity of SESM incorporated in the mucoadhesive layer. To achieve this aim, CHX, in the form of digluconate salt, was selected as a model drug, since its local delivery in the mouth may enhance the treatment of oral diseases if its concentrations in the saliva can be maintained at effective, but not noxious, levels for a prolonged period [43].

A supporting layer between the two external layers was formulated from a polymeric mixture of HPC and a film-forming agent with a texture function, which helped the fabrication process, improved the mechanical profile of the final dosage form and hindered the erosive action of the saliva.

The most interesting multilayer films thus obtained were characterized for their physico-chemical properties like mucoadhesion, mechanical strength and drug release control. The achieved preliminary results show that this platform might be successfully employed for the local treatment of oral lesions.

## 2. Materials and Methods

### 2.1. Materials

The following materials were used: hydroxyethylcellulose (HEC) (Natrosol 250 L, G and HHX Pharm grades, Ashland, Wilmington, DE, USA); hydroxypropylcellulose (HPC) (Klucel GF Pharm and MF Pharm grades, Ashland, Wilmington, DE, USA); hydroxypropylmethylcellulose (HPMC) (Methocel E15 Premium LV, K15M Premium, Dow, Midland, MI, USA; Benecel K100LV PH PRM and K750 PH PRM grades, Ashland, Wilmington, DE, USA); sodium carboxymethylcellulose (CMC) (Blanose 7LP EP and 7M8SF PH grades, Dow, Midland, MI, USA); methylcellulose (MC) (Benecel A4M and A15 LV grades, Ashland, Wilmington, DE, USA); polyvinylalcohol (PVA) (18-88 and Parteck SRP 80 grades, Merck KGaA, Darmstadt, Germany); polyvinylpyrrolidone (PVP) (Kollidon 30 and 90 F grades, BASF, Ludwigshafen, Germany); vinylpyrrolidone-vinyl acetate copolymer (PVP-VA) (Kollidon VA64 grade, BASF, Ludwigshafen, Germany); polyethyleneoxide (PEO) (Polyox WSR N-80 NF grade, MW 200 kDa, Dow, Midland, MI, USA; polyethyleneoxide MW 1000 and 2000 kDa, Sigma Aldrich, Saint-Louis, MO, USA); high methoxyl pectin citrus (HM, degree of methoxylation 61%, Alfa Aesar, Haverhill, MA, USA); carbopol 971P NF (Lubrizol, Wickliffe, OH, USA); poloxamer P407 (Kollyphor P407, BASF, Ludwigshafen, Germany,); sodium alginate from kelp (viscosity of 2% solution = 237 cPs, Sigma Aldrich, Saint-Louis, MO, USA); hydroxypropyl pea starch (HPSP) (Lycoat RS720, Roquette, Cassano Spinola, Italy); bovine hide gelatin (GEL) (175 H 30, gel strength 166 g Bloom, Rousselot, NM Son, The NederlandsNLNetherlands); bovine collagen peptide (BCP5) (Peptan B 5000 HD, 5 kDa, Rousselot, NM Son, The Netherlands); chlorhexidine digluconate (CHX, 20% *w*/*v* aqueous solution); mucin from porcine stomach type II; disodium hydrogen phosphate dihydrate; potassium dihydrogen phosphate anhydrous and sodium chloride (Sigma-Aldrich, Saint-Louis, MO, USA); Columbia Agar plates (Oxoid, Basingstoke, UK); Triptic Soy Broth (Biolife, Milano, ItalyT); Roswell Park Memorial Institute (RPMI) medium, Dulbecco’s Modified Eagle Medium (DMEM) and Fetal Bovine Serum (FBS) (Gibco, Grand Island, NY, USA); and lipopolysaccharide (LPS, Invitrogen, Waltham, MA, USA).

### 2.2. Preparations

#### 2.2.1. Preformulation Study

Pure polymer aqueous solutions (5% *w*/*w*) were prepared by dispersing the polymers in deionized water at room temperature (HPMC K100LV and K750 PH, MC, PVA, PVP, PVP-VA, PEO and HPSP) or at 37 °C (HPC, HEC, HPMC E15 and K15M, CMC, pectin, carbopol 971P, poloxamer P407, sodium alginate and bovine collagen peptide), depending on the optimal solubilizing conditions as reported in the polymer brochures or in the literature. To remove air bubbles from the solutions, they were maintained at room temperature at rest for at least 2–3 h before casting.

For polymer solution, 0.5 g were cast on polystyrene plate lids (mold diameter = 23 mm) and dried at 45 °C until they presented a constant weight (normally 1 h) in a thermostated oven; then, the obtained films were peeled off. Only the ones that appeared adequate in terms of flexibility and mechanical resistance were evaluated for in vitro adhesion and mechanical properties.

#### 2.2.2. Preparation of Soluble Eggshell Membrane (SESM)

SESM was prepared according to a method previously described in the literature [35], with slight modifications.

ESM were separated from the eggshells by manually peeling them off, placing them in 5% EDTA (2,2′,2″,2‴-(ethane-1,2-diyldinitrilo)tetraacetic acid) solution for 1 h to remove traces of eggshell or calcite, then washing them with milliQ water thrice and lyophilizing them. After this, approx. 0.4 g of lyophilized eggshell membrane were hydrolyzed with 40 g of 3 N NaOH aqueous solution at 37 °C under 300 rpm orbital shaking until complete solubilization was observed (3–4 h).

The solubilized eggshell membrane solution was neutralized with 3 N HCl aqueous solution and aliquots weighing 3 g were ultrafiltered on a Millipore 10 kDa MWCO filter at 6000× *g* for 10 min at 15 °C. The retentate was dissolved in milliQ water and ultracentrifuged several times until the solution was completely desalted (verified by the silver nitrate assay). Finally, the retentate was dissolved with milliQ water and lyophilized. The yield was estimated by comparing the lyophilized SESM weight to the initial weight of lyophilized ESM used.

#### 2.2.3. Preparation of Monolayer Mucoadhesive Films

SESM was added to HPC GF, which had resulted as the best film-forming polymer with mucoadhesive properties in the preformulation study, in the following different percentage ratios: 100/0, 80/20, 70/30 and 50/50 (%SEMS/%HPC GF *w*/*w*). The composition that resulted in the film with optimal surface texture and homogeneity (which was 70/30% *w*/*w*) was selected for further characterizations, which were undertaken in comparison to neat HPC GF films and to films where SESM was replaced with the biopolymer GEL or collagen peptide BCP5 (MW 5 kDa).

The casting solutions including 3.5% *w*/*w* HPC GF and 1.5% *w*/*w* biopolymer were prepared through the dispersion of accurately weighed powders in appropriate volumes of deionized water at 37 °C under continuous stirring. Then, 0.5 g of solution were cast and dried at 45 °C until they reached a constant weight. The prepared films (weighing approx. 25 mg) were peeled from the mold and evaluated for in vitro adhesion and mechanical properties.

#### 2.2.4. Preparation of Bilayer Mucoadhesive Films

For bilayer films, firstly, the selected polymer mixtures were dispersed in appropriate volumes of deionized water under magnetic stirring at 37 °C for 1 h. After complete homogenization, a double casting method was used: the supporting layer was prepared by casting 0.5 g of an aqueous solution of 3.5% *w*/*w* HPC GF + 1.5% *w*/*w* biopolymer or film-forming polymer (specifically, GEL, pectin, sodium alginate or CMC 7LP) and drying it at 45 °C; the mucoadhesive layer was obtained through the subsequent casting of 0.5 g of an aqueous solution of 3.5% *w*/*w* HPC GF + 1.5% *w*/*w* SESM, and drying at 45 °C.

The prepared films were peeled from the mold and evaluated for their mechanical properties.

#### 2.2.5. Preparation of Trilayer Mucoadhesive Films

For trilayer films, firstly, the selected polymer mixtures were dispersed in appropriate volumes of deionized water under magnetic stirring at 37 °C for 1 h. After complete homogenization, 0.5 g of the solutions containing 5% *w*/*w* HPC GF, or 2.5% *w*/*w* HPC GF + 2.5% *w*/*w* HEC G or 2.5% *w*/*w* HPC G + 2.5% *w*/*w* HPMC K750 and 1% of CHX were cast and dried at 45 °C (creating the drug delivery layer). The supporting layer was obtained by casting 0.5 g of a polymer solution containing 3.5% *w*/*w* HPC GF + 1.5% *w*/*w* GEL on the first layer and drying it. Finally, the mucoadhesive layer was obtained by casting 0.5 g of a solution of 3.5% *w*/*w* HPC GF + 1.5% *w*/*w* SESM and drying it. The obtained trilayer films were peeled from the mold and evaluated for in vitro and ex vivo mucoadhesion, mechanical properties, swelling and erosion index, in vitro and in vivo residence time and their CHX release profile.

### 2.3. Film Characterization

#### 2.3.1. Morphological Data and Surface Evaluation

The films (*n* = 5) were weighed on a digital analytical balance. Thickness was measured with a digital caliper in five random positions on the film, and the average value was determined.

The appearance of the films was evaluated using optical microscopy (Alphaphot-2YS2, Nikon, Tokyo, Japan) and a 10× objective. Illustrative digital images were acquired at the same time using Moticam 5 equipment (Motic, Hong Kong).

#### 2.3.2. In Vitro Mucoadhesion Studies

In vitro mucoadhesion was assessed by studying the detachment of the film from a substrate mimicking the mucosal surface (250 mg porcine mucin tablets prepared using a 13 mm mold applying a compression force of 6 tons for 5 min through a hydraulic press). A material testing machine (LRX model, Lloyd Instruments, Bognor Regis, UK), equipped with a 20 N load cell, was used.

The tested film was pasted on the upper support connected to the machine with cyanoacrylate glue; the porcine mucin disc was glued on the lower support and wetted with 50 μL of pH 6.8 PBS. Then, the upper support was lowered to place the film in contact with the mucin tablet, and a preload of 1 N was applied for 10 min, before the adhesive force was continuously measured by pulling on the upper support at a velocity of 0.1 mm/s.

Maximum detachment force (MPa) is the ratio between the maximum applied force necessary to detach the patch from the mucin substrate and the area of the film in contact with the substrate; the total work of adhesion (mJ) is the area delimited by the adhesion test curve and the *x*-axis [44,45].

The results are expressed as the mean of 3 determinations ± SD.

#### 2.3.3. Ex Vivo Mucoadhesion Studies

This test was performed with the above-mentioned material testing machine equipped with a 20 N load cell, using the porcine buccal mucosa, chosen due to its resemblance to human buccal mucosa [46,47].

The film was pasted on the upper support of the machine, while a piece of biological tissue (24 × 24 mm square) was glued on the lower support and wetted with 50 μL of 10% *w*/*v* porcine mucin aqueous solution; then, by lowering the upper support and applying a preload of 1 N, the film made contact with the biological tissue. After 10 min, the adhesive force was measured by pulling on the upper support at a speed of 0.1 mm/s. The maximum detachment force and work of adhesion were obtained from the output graph. The results are expressed as the mean of 3 determinations ± SD.

#### 2.3.4. Mechanical Properties

Yield strength (YS), Young’s modulus (YM) and elongation at break (EB) were studied using the testing machine, equipped with 20 N or 100 N load cell and mechanical grips, one of which was mounted on the movable arm and one of which was fixed. After placing the film (10 × 10 mm) between the two grips, the movable arm was lifted at a speed of 1 mm/min, applying a progressively increasing tensile stress until the film broke. The instrument continuously registered the force applied, generating a displacement/force graph. In the resulting peak, the maximum force value was marked as peak stress. By dividing the values of displacement by the initial film length, values of strain were calculated; by dividing the force values by the film area, stress values were calculated and a stress–strain curve was obtained.

The mechanical properties were calculated using the following equations:

Yield strength (MPa) = peak stress/film cross-sectional area

Young’s modulus (MPa) = slope of the linear part of the stress/strain curve

Elongation at break (%) = increase in length at break/initial film length × 100

Puncture strength was measured as the force applied by the tensile machine to let a probe with hemispherical end (5 mm diameter) pass with a velocity of 1 mm/min through the film fixed on a support with a circular hole in the center.

The results are expressed as the mean of 3 determinations ± SD.

#### 2.3.5. Swelling and Erosion Index

Due to the gelled films’ fragility, measuring their swelling properties in pH 6.8 PBS was challenging, since their recovery from the Petri dish and weighing were impossible. Therefore, the swollen films were directly weighed in their container, after removing the excess of liquid around them.

For this purpose, each film was weighed (W1) and then placed on a pre-weighed 35 mm diameter Petri dish. Then, the films were covered with 4 mL of PBS and maintained for 30, 60, 120 and 180 min at 37 °C. Then, after carefully removing the excess liquid around the gelled films with a pipette, the Petri dishes were weighed again to determine the weight of the swollen film (W2), and the swelling index (SI) was calculated. Then, the films were dried for 1 h at 60 °C in a ventilated oven to measure the residual weight (W3), and the erosion index (EI) was calculated using the following equations:

SI (%) = (W2 − W1)/W1 × 100

EI (%) = (W1 − W3)/W1 × 100

#### 2.3.6. In Vitro Residence Time

This test was performed by pressing the films against a glass plate hung on the movable support of an appropriately modified disintegrating compendial apparatus, moving vertically into and back out of 900 mL of PBS (pH 6.8) maintained at 37 °C; the time taken for the film to detach or completely erode was recorded. The results are expressed as the mean of 3 measures ± SD.

#### 2.3.7. In Vivo Residence Time

This study, involving the 8 authors as healthy volunteers, was carried out by pressing a film not including CHX on the gingival mucosa above the canine tooth for 10 s. The residence time was measured as the time taken before the complete detachment or erosion of the film from the mucosa occurred. The results are reported as the mean of the 5 determinations ± SD. All participants gave their consent to participate in the test after being informed about the composition of the film and the test procedure.

#### 2.3.8. In Vitro Drug Release Studies

A modified version of a previously used home-made device [48] was employed to perform the in vitro dissolution test. First, a plunger of a 50 mL syringe was inserted into the polypropylene tube deprived of its Luer extremity in inverted position to exploit the plunger flange as support for film attachment. An accurately weighed film was then glued to the flange, and the plunger was moved to place the film in contact with 10 mL of pH 6.8 PBS simulating human saliva [49], so that the disc was just below the liquid surface. The dissolution medium was maintained under soft orbital shaking (200 rpm) in a Peltier chilling–heating dry bath (Torrey Pines Scientific Inc., Carlsbad, CA, USA), at a constant temperature of 37.0 ± 0.2 °C. At each sampling time (set at 30 min, 1, 2, 3 and 4 h), 3 different devices were used, so that each time point in the release curves resulted from the mean of 3 values. The concentration of CHX in the dissolution medium was determined spectrophotometrically (Hewlett Packard 8453 UV spectrophotometer, Palo Alto, CA, USA) at 254 nm. The drug released was expressed as the *w*/*w* percentage of the drug loaded.

#### 2.3.9. In Vitro Sterility Test

During the manufacturing process, the films can be easily contaminated, possibly leading to the further infection of wound sites. Therefore, a sterility test was carried out on the films, employing the two following methods:

(i). Incubation on rich medium agar plates: each sample, placed aseptically on Columbia Agar plates, was incubated overnight at 37 °C and 23 °C. Then, the plates were carefully examined for visible microbiological growth and further incubated for 48 h before the evaluation of microbiological contamination continued.
(ii). Incubation in nutrient broth medium: each sample, placed aseptically in 5 mL of Triptic Soy Broth, was incubated overnight at 37 °C and 23 °C. Afterwards, 200 μL from each culture broth, after seeding on Columbia Agar plates, were incubated at 37 °C and 23 °C overnight; the plates were then carefully evaluated for visible growth. Subsequently, broth samples were stored at 37 °C and 23 °C for a further 14 days, then re-plated onto Columbia Agar plates and re-incubated for 24 h, as previously described.

##### Aseptic Preparation of Final Dosage Form

For the trilayer film formulations mentioned in Section 2.2.5, five units were tested for sterility. Initially, appropriate amounts of the various components were weighed, placed in Petri dishes and UV-sterilized overnight under a vertical laminar flow hood. The casting solutions were prepared by adding sterile water and appropriate amounts of powders into individual sterile vials under continuous stirring in a laminar flow hood for 1 h. Then, 0.5 mL (equivalent to 0.5 g) of the solutions corresponding to the backing layers were cast on a sterile polystyrene mold and left dry in the laminar flow hood for 4 h. Also, the supporting and mucoadhesive layers were obtained by casting 0.5 mL of the polymer solutions, which were left to dry at room temperature for 4 h in the laminar flow hood. The dried films were exposed overnight to UV light, and then, using sterile tweezers, carefully collected and placed in self-lock bags for sterility testing.

### 2.4. Biological Tests

#### 2.4.1. Cell Cultures

Human leukemia monocyte THP1 cell line (THP-1) and human keratinocyte HaCat cells were obtained from the Biological Bank and Cell Factory (IRCCS AOU San Martino-IST, Genova, Italy).

#### 2.4.2. Biological Study of SESM Cell Compatibility and Anti-Inflammatory Activity

The MTS assay (CellTiter 96^®^ Aqueous One Solution Cell Proliferation kit, Promega, Madison, WI, IT) was used to evaluate cell viability, and the gene expression of IL-8, a chemokine produced by macrophages, was quantified using RT-PCR. LPS was used as a positive control for monocyte activation, being one of the most potent innate immune-activating stimuli known.

Human leukemia monocyte THP1 cell line (THP-1) was cultured in standard culture conditions (37 °C and 5% CO_2_) with RPMI-1640 (Euroclone^®^, Milano, Italy) and supplemented with 10% (*v*/*v*) FBS (Euroclone^®^) and 2 mM L-glutamine (Euroclone^®^), without antibiotics or antifungal supplements. THP-1 cells were sub-cultured every 3–4 days when the cell density exceeded 1 × 10^6^ cells/well. All cultures were found to be mycoplasma-free during regular checks with the Reagent Set Mycoplasma Euroclone (Euroclone^®^).

For MTS and RT-PCR, THP-1 cells were seeded in 96-microwell plates (approx. 3 × 10^4^ cells/well) and 75 cm^2^ tissue flasks (approx. 5 × 10^5^ cells/flask), respectively. After 24 h, the cells were exposed to LPS alone, SESM + LPS and SESM alone, at a concentration of 1 and 3 mg/mL for SESM and of 5 µg/mL for LPS in complete growth medium. At 48 and 72 h, for the MTS assay, the 490 nm absorbance of formazan was measured in a microtiter plate-reader (Uniskan II, Labsystem Diagnostics, Vantaa, Finland); at 6 and 24 h, RT-PCR was carried out on the controls, on the cells exposed to LPS alone, SESM 3 mg/mL and LPS + SESM 3 mg/mL.

The effect of each treatment was extrapolated and expressed as percentage vs. control (i.e., untreated) cultures and presented as the mean ± SD of 3 independent experiments, with 3 replicates performed for each condition.

#### 2.4.3. SESM Wound Healing Assay

To evaluate cell migration in vitro, human keratinocyte HaCat cells were seeded in 6 microwell plates (approx. 8.5 × 10^5^ cells/well) and grown in DMEM medium without antibiotics or antifungal supplements, before being added to 10% FBS at 37 °C with 5% CO_2_ until confluence was reached. Later on, a scratch was made using a p200 pipet tip, and, to smooth the edge of the scratch and remove the debris, a gentle washing with PBS was carried out [50]. The cells were then cultured with standard medium containing 10% FBS (as a positive control) or with medium alone (as a negative control) or with 3 mg/mL SESM solution in DMEM Complete Medium, containing 10% FBS. Cell images were acquired at 0 h and after 24 and 48 h using a phase-contrast microscope, and the distances of the scratched region contours were measured using Image J software, 1.54j version. The wound closure % was calculated as follows:

Wound closure % = (W0 − Wt)/W0 × 100%

where W0 denotes the initial width of the scratch and Wt denotes the scratch width t hours after the test start. Each experiment was performed with 4 replicates.

#### 2.4.4. In Vitro Film-Blood Compatibility Test: Whole Blood Clotting

For this test, neat PTFE discs were used as the negative control and as supports for 3 mg/mL SESM solution in PBS, and a medical gauze was used as the positive control. The blood samples used were obtained from the authors.

A method previously described in the literature was applied to perform this test [15]. The films to be tested were placed into plastic dishes and were pre-warmed to 37 °C. Then, 0.2 mL of whole blood [collected in BD vacutainer^®^ glass blood collection tubes with ACD (Anticoagulant Citrate Dextrose) solution type A (citric acid 8 g/L, trisodium citrate 22 g/L, dextrose 24.5 g/L)] were dripped onto their surface, and 50 μL of 0.2 M CaCl_2_ solution were added in order to induce coagulation. The plastic dishes were subsequently incubated for 10 min at 37 °C, and the red blood cells (RBCs) not trapped in the clot were hemolyzed with 10 mL of deionized water, following centrifugation at 100× *g* for 30 s. Finally, 5 mL of supernatant were transferred carefully into a tube and diluted with 20 mL of pure water.

The blood clotting index (BCI) was calculated as follows:

BCI = Ds/D0

where Ds denotes the absorbance of the solution of hemoglobin released by the untrapped RBCs measured at 542 nm and D0 denotes the absorbance of whole blood (0.2 mL) hemolyzed with of deionized water (25 mL) measured at 542 nm.

The results are expressed as the mean of 3 determinations ± SD.

#### 2.4.5. Statistical Analysis

A one-way analysis of variance (ANOVA) was used to compare the data regarding the in vitro mucoadhesion capacity and the mechanical properties of trilayer films. Using a Bonferroni’s test, a post hoc comparison test of the means of individual groups was carried out. The level of significance was set at *p* = 0.05. The statistical evaluation of data was performed using Systat software, version 13.1.

## 3. Results and Discussion

### 3.1. Preformulation Study

The rationale behind conducting the preformulative study was to select the best polymers with film-forming properties featuring aqueous solubility, good flexibility without plasticizers, appropriate mechanical and mucoadhesive characteristics and availability in a wide range of viscosities.

The solvent casting method was selected to prepare monolayer films, since it is a widely used manufacturing process, which is well-known for the low cost that the system setup requires at a research laboratory scale and its relative simplicity [32].

Monolayer films were prepared by casting single aliquots of polymeric solutions on a polystyrene mold, so as to know exactly the amount of polymer included in the film. Moreover, films produced by this method show good uniformity of weight and thickness [44].

The list of polymers cast is reported in Table S1 of the Supplementary Materials.

Besides some polymer dispersions that were too viscous to cast, and some that gave films undetachable from the polystyrene surface, several water-soluble, natural film-forming polymers, like sodium alginate and pectins, as well as some cellulose-based polymers like CMC, low viscosity grades of PVP and poloxamer, resulted in rigid films that were not suitable for direct application on the anatomic irregularities of gingival mucosa. Other ones, such as PVP K30 or poloxamer, resulted in brittle films that were not able to withstand manipulation. The films obtained from PVP K90, PEO and HPSP were too thin (<0.05 mm) and, therefore, were not considered for further studies.

Among the tested polymers, cellulose derivatives like HPC, HPMC and HEC, and synthetic compounds like PVA, produced films with good flexibility without the use of plasticizers and, for this reason, were selected as possible candidates for the following properties of the preformulative evaluation, including: the flowability of the casting solution, the structure and appearance of the films, the detachability from the mold, the in vitro adhesion on a mucin tablet and the tensile stress–strain behavior. The results are reported in Table 1.

On the basis of the obtained results, films made of HPC GF and the two grades of PVA showed higher detachment force than the ones made of HEC G and HPMC K750 (*p* < 0.05), whereas the work of adhesion was tendentially but not statistically higher for films made of HPMC E15, HPMC K100LV and PVA SRP 80, compared to HEC G-based films.

HPMC K750 and E15 showed significantly higher YS than the other polymers (*p* < 0.05). As shown by YM, films made of pure HPC GF showed the most elastic behavior (*p* < 0.05), which correlated well with the results of EB. Surprisingly, despite their low YM, films made with PVA 18-88 showed poor EB.

In conclusion, HPC GF was chosen as the best candidate for the mucoadhesive layer, which will be in contact with the mucosa, as it showed good mucoadhesiveness and high mechanical resistance.

### 3.2. Biological Study on SESM

#### 3.2.1. SESM Preparation

SESM was prepared by applying an optimized protocol that involved the initial removal of calcium carbonate from eggshell membrane, its solubilization in sodium hydroxide aqueous solution and its neutralization and desalting. The yield was approx. 35% of the weight of the initial membrane.

#### 3.2.2. Cell Compatibility and Anti-Inflammatory Activity

**Figure 1:**
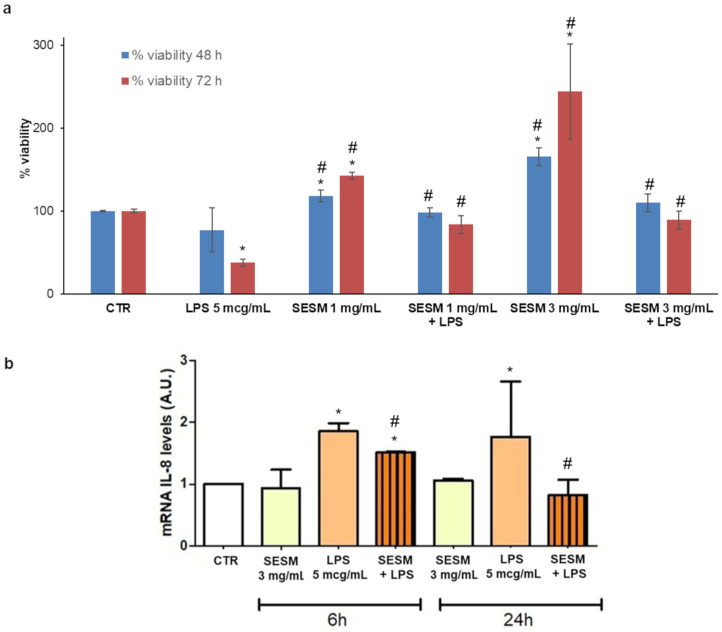
(**a**) Viability index extrapolated from MTS assay at 48 and 72 h; (**b**) IL-8 gene expression at 6 and 24 h (values represented as mean ± SD, *n* = 3; the symbol (*) denotes statistical significance from the corresponding control (CTR); the symbol (#) denotes statistical significance from the corresponding treatment with LPS (*p* < 0.05)).

The viability and proliferation of THP-1 cells in the presence of SESM were determined using the MTS assay, while the expression of IL-8 chemokine gene was measured using RT-PCR. A decrease in the viability index, extrapolated from the MTS assay, occurred with a time-proportional trend after exposure to LPS. SESM significantly increased the viability, not only in comparison to inflammation-induced cells, but also to the control untreated cells in a concentration-dependent fashion, as shown by viability index values, which more than doubled if a 3 mg/mL SESM concentration was used. Even when in the presence of LPS, SESM was able to preserve a cell viability comparable to the one of the untreated group, somehow “neutralizing” LPS’s effects (Figure 1a). This proliferation enhancement effect of ESM, observed and investigated by several authors, represents a key feature for the potential application of this biomaterial in wound healing and other biomedical applications [51,52].

After adding LPS, THP-1 activation was assessed in terms of the modulation of IL-8 gene levels, which are representative of proinflammatory chemokine synthesis. Indeed, it was observed that, just after 6 h exposure to LPS alone, the chemokine gene levels increased, remaining constant for up to 24 h; SESM exerted a protective effect against inflammation (Figure 1b). This result is comparable to the one observed by Shi et al. [53], who stimulated Caco-2 cells using TNF-α and measured a significant inhibition of IL-8 secretion when the cells were exposed to SESM 1 mg/mL; also in that case, SESM showed no effect on IL-8 secretion in the absence of prior TNF-α stimulation.

#### 3.2.3. Wound-Healing Assay

**Figure 2:**
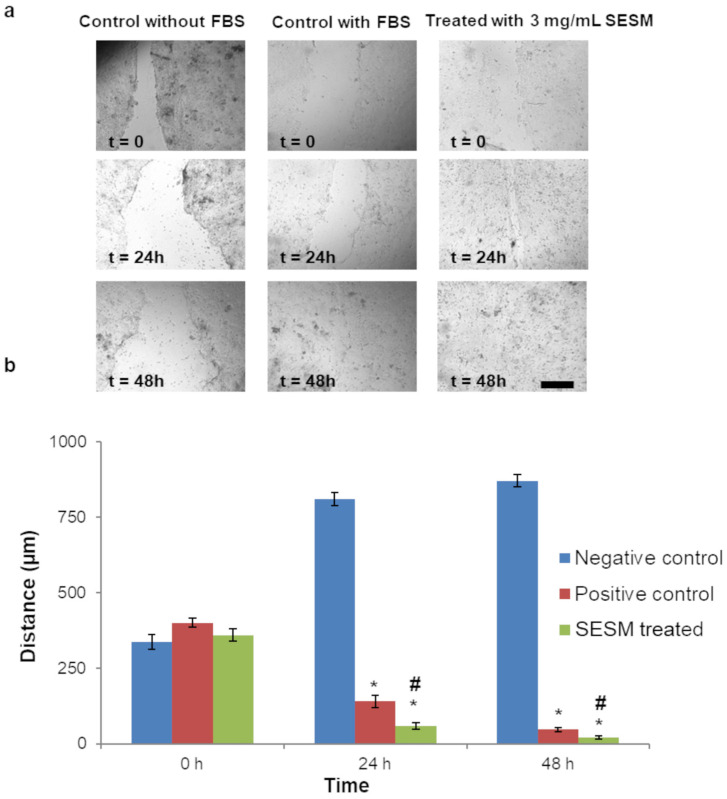
(**a**) Effect of SESM on HaCaT keratinocyte migration at 0, 24 and 48 h; (**b**) quantitative analysis of wound-healing effect in cell cultures incubated with FBS (positive control), without FBS (negative control) and SESM (values represented as mean ± SD, *n* = 4; the symbol (*) denotes statistical significance from the negative control; the symbol (#) denotes statistical significance from the positive control (*p* < 0.05); scale bar = 200 µm).

The cell wound closure assay was used to assess the ability of keratinocytes to migrate and subsequently close a wound previously made in a plate of cells at confluence. In the presence of 3 mg/mL SESM in DMEM, the migration of HaCaT cells into the wounded area was clearly enhanced (Figure 2a,b). In particular, the effect was evident after 24 h, when the SESM-treated cells showed a wound closure of 84 ± 12% (positive control 30 ± 9%). The wound healing properties of eggshell membrane have already been highlighted (for example in [54]), but the innovative aspect of our result is represented by the use of the soluble form of eggshell membrane, which grants better bioavailability to the eggshell membrane components, thereby promoting wound closure.

### 3.3. Formulation Study of Mucoadhesive Layer

**Figure 3:**
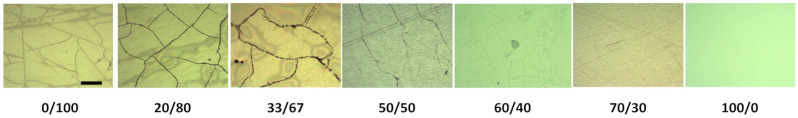
Surface morphology of films with different HPC GF/SESM ratios (scale bar 100 µm).

The compatibility of HPC GF with SESM in different ratios was first tested using optical microscopy to evaluate the surface texture and homogeneity of the films. HPC GF maintained its film-forming ability, but its mechanical properties worsened when ≥50% SESM was added (Figure 3). Hence, mixtures of HPC G + SESM 70/30% *w*/*w* were used to prepare mucoadhesive films.

On the other hand, no significant differences in the morphological characteristics of the monolayer films including different amount of SESM were highlighted: the films weighed between 25.5 and 26.2 mg and were approx. 52–60 µm thick (*n* = 5).

**Figure 4:**
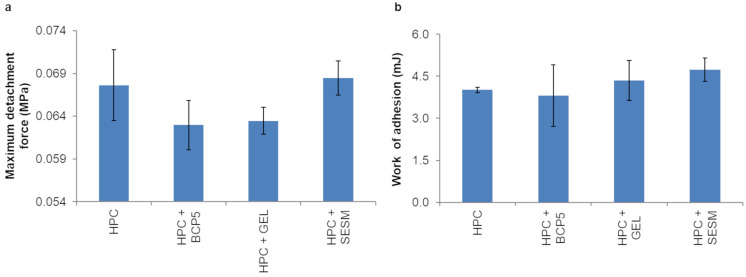
Maximum detachment force (**a**) and work of adhesion (**b**) of films made of HPC G with/without biopolymers tested on mucin tablet (values represented as mean ± SD, *n* = 3).

Figure 4 illustrates the adhesive properties of monolayer films made of HPC GF with and without biopolymers (SESM was compared to bovine collagen peptide 5 kDa and gelatin). It is evident that the addition of SESM had no negative effect on the detachment force of HPC GF (*p* > 0.05) and, furthermore, slightly increased the work of adhesion (even though the difference was not significant). On the contrary, the other biopolymers tested seemed to have a tendential negative effect on maximum detachment force and no considerable effect on work of adhesion (*p* > 0.05).

**Figure 5:**
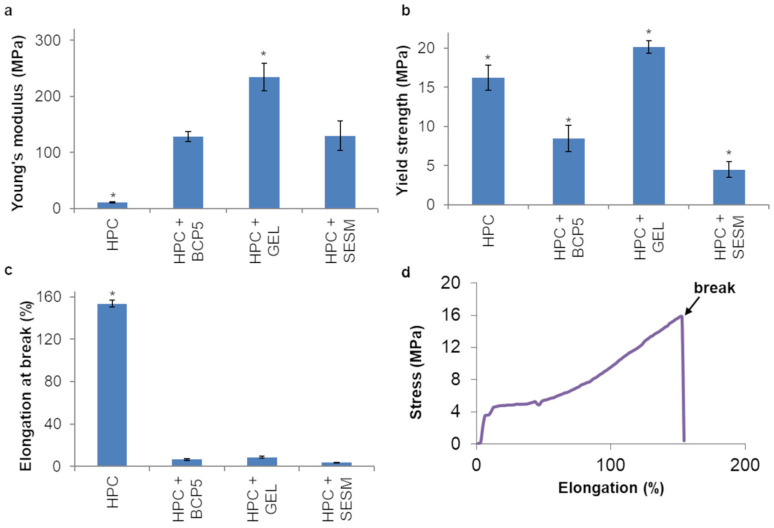
YS (**a**), YM (**b**), EB (**c**) and an exemplificative elongation/stress curve (**d**) of monolayer films (values represented as mean ± SD, *n* = 3; the symbol (*) denotes statistical significance from the other compositions (*p* < 0.05)).

The mechanical properties of the monolayer films are shown in Figure 5. The addition of biopolymers to HPC GF had a significant (*p* < 0.05) negative effect on the YS, except GEL, which, on the contrary, made the films more resistant than the HPC GF ones (*p* < 0.05).

Also, all the tested biopolymers increased the rigidity of the HPC GF films, as confirmed by the increases in YM and the reductions in EB (*p* < 0.05).

From the studies performed on the first layer, it was evident that SESM did not cause any negative effect on adhesion, despite weakening the mechanical properties of the HPC GF polymer; actually, this effect was expected, as it had already been evidenced by other authors for films including a different polymer [55]. On the basis of the previous considerations, SESM was chosen as biopolymeric component of the mucoadhesive layer.

**Figure 6:**
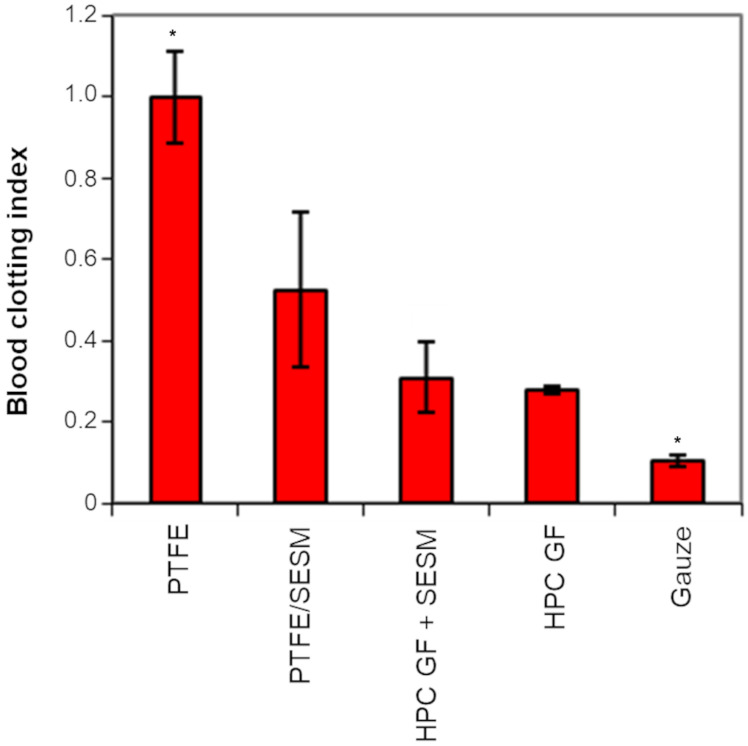
Blood clotting index. PTFE/SESM denotes SESM deposited on PTFE (values represented as mean ± SD, *n* = 3; the symbol (*) denotes statistical significance from the other compositions (*p* < 0.05)).

The first layer applied on the wound must be protective and not inhibit blood coagulation; therefore, the absence of any interference with blood clotting of the selected mucoadhesive layer, in comparison to a neat HPC GF film and to SESM solution alone on PTFE (used as negative control), was evaluated by measuring the BCI, whose low values indicate a better blood clotting compatibility (the positive control for this was a gauze). From Figure 6, it is clear that SESM did not have a negative effect on blood clotting.

### 3.4. Formulation Study of Bilayer Films

As the HPC GF/SEMS film was relatively weak, a supporting layer was considered necessary for giving texture to the entire multilayer structure.

To this aim, bilayer films including the mucoadhesive HPC GF + SESM 70/30 layer were prepared. The second layer was made by 70/30% *w*/*w* polymer combinations of HPC GF with those polymers that had produced rigid films either when combined with it (GEL, as shown in Section 3.4) or when used alone in the preformulation study (pectin HM, sodium alginate or CMC 7LP). The association of these polymers to HPC GF was considered beneficial also in preventing the diffusion of active substances loaded in the external layer towards the lesion on the gum.

The resulting films weighed approx. 51.5 mg and were 115 µm thick, independently of the composition (*n* = 5).

On the bilayer films, only the mechanical properties were tested, to verify whether the addition of the supporting layer might improve the poor resistance of the mucoadhesive layer.

**Figure 7:**
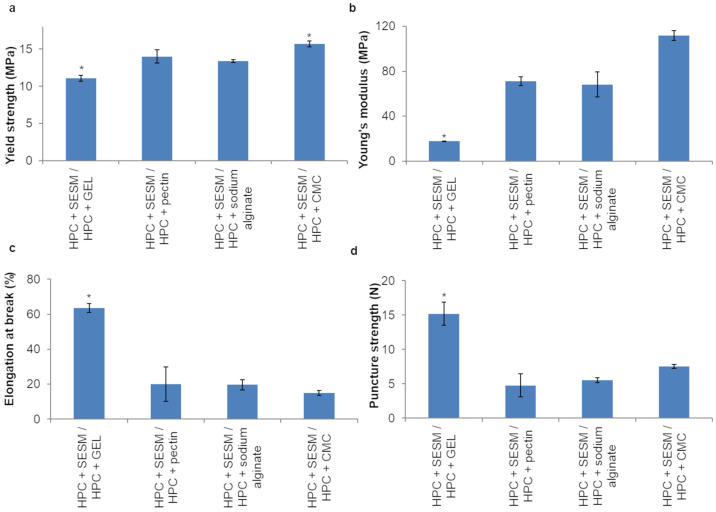
YS (**a**), YM (**b**), EB (**c**) and puncture strength (**d**) of bilayer films (values represented as mean ± SD, *n* = 3; the symbol (*) denotes statistical significance from the other compositions (*p* < 0.05)).

The YS of the films whose supporting layer contained pectin, sodium alginate and CMC was slightly higher than that of the HPC + GEL films (Figure 7a), but these films were too rigid and, consequently, showed a low EB compared to the HPC + GEL films, and the difference was statistically significant (*p* < 0.05, Figure 7c). In addition, the results of puncture strength showed that the films containing HPC and GEL were ruptured at a higher load than films containing pectin, sodium alginate and CMC 7LP (*p* < 0.05), and this behavior might be related to the elastic nature of this polymer.

In the end, based on the results obtained, HPC GF + GEL 70/30 was selected as the composition for a potential supporting layer.

### 3.5. Formulation Study of Trilayer Films

**Figure 8:**
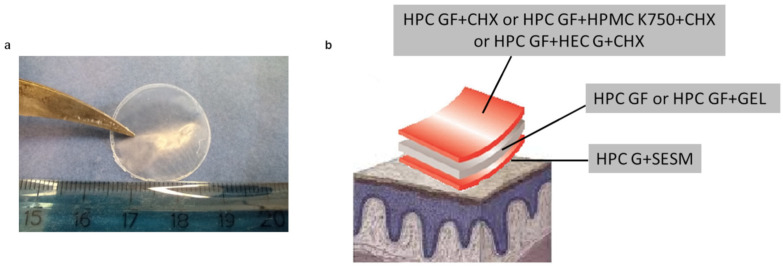
Image (**a**) and scheme (**b**) of a trilayer film obtained by casting.

The third layer, or backing layer, of the trilayer films (Figure 8) was conceived to control the release of an antimicrobial drug. To this aim, HPMC K750 and HEC G were selected, since they are film-forming agents but are also known for their ability to control the drug release, in comparison to a layer including only HPC GF. Moreover, HPMC, due to its molecular weight, might play a role in extending the erosion time of the film and prolonging its residence time, as also observed by Wojtyłko et al. [56]. Conversely to the fast dissolving films described in the literature, in which low-viscosity-grade HPMC with the eventual addition of disintegration agents is required, in formulations designed for an extended stay in the oral cavity, a higher polymer viscosity is necessary, so HPMC types characterized by a higher viscosity are preferred [57].

This third layer was loaded with a total amount of 5 mg of CHX; this small amount was chosen because it is advisable not to expose the lesion to a high CHX concentration, due to its potential interference with the healing properties of the SESM in the mucoadhesive layer. On the other hand, considering that CHX MIC values for most pathogens are lower than 50 µg/mL [58], this oral concentration would be effective in reducing the bacterial load in the oral cavity.

The trilayer films were prepared using multiple casting and evaluated for adhesion, mechanical properties, swelling and erosion index, residence time and in vitro drug release rate.

The morphological characteristics of the trilayer films studied are reported in Table 2. Moreover, the presence of CHX in the film did not cause the appearance of any surface roughness, as visible in Figure 8a, contrarily to other film formulations reported in the literature [59].

**Figure 9:**
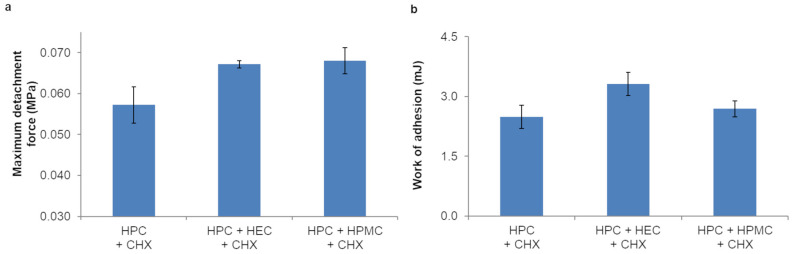
Maximum detachment force (**a**) and work of adhesion (**b**) of trilayer films (only the composition of the backing layer is indicated) tested on mucin tablet (values represented as mean ± SD, *n* = 3).

As for in vitro adhesion properties of the prepared trilayer films, no significant differences (*p* > 0.05) in the evaluated parameters were found among the films (Figure 9).

However, the values of detachment force and work of adhesion of the trilayer films whose backing layer included just HPC were lower than the ones measured for HPC GF + SESM monolayer films. This might be explained by the fact that mucoadhesive polymers must reach a critical degree of hydration for optimum swelling and mucoadhesion [60]. Two additional external layers might attract water from the mucoadhesive one, slightly reducing its hydration and its adhesivity; however, when HEC or HPMC are added to the backing layer, they might make the layer more impermeable, reducing the amount of water uptaken by the external layers of the film, and thus restoring the original adhesiveness of the mucoadhesive layer. This hypothesis may be corroborated by the observation of Juliano et al., who investigated the swelling behavior of double layer films composed of an external HPMC layer and an internal alginate layer by alternately exposing the cellulosic layer or the polysaccharidic layer to the medium; in particular, they observed low hydration and swelling of the double layer film when the HPMC layer was exposed to the medium. Hence, HPMC was supposed to hinder the hydration of the underling alginate layer [61].

**Figure 10:**
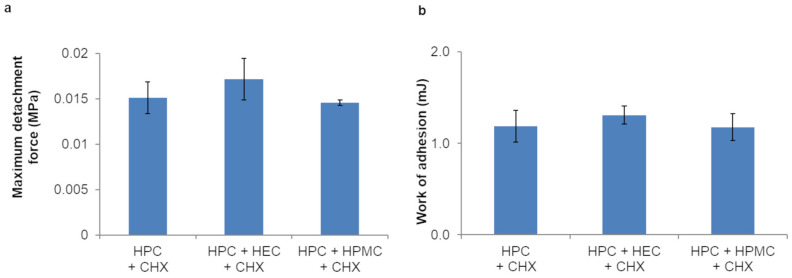
Maximum detachment force (**a**) and work of adhesion (**b**) of trilayer films (only the composition of the backing layer is indicated) tested on porcine mucosa (values represented as mean ± SD, *n* = 3).

The results of ex vivo mucoadhesion studies performed on porcine buccal mucosa (Figure 10) showed that the maximum detachment force and the work of adhesion slightly but not significantly (*p* > 0.05) increased for films containing HPC GF + HEC as third layer, compared to the other trilayer films.

The selection of the appropriate biomaterial to use to measure the adhesion of the developed formulation is a considerable challenge in mucoadhesion studies, and the different choices of the researchers often hamper the comparison with the data in the literature. However, porcine and bovine oral mucosae are frequently used, since, differently from most rodents, they exhibit non-keratinized membranes similar to humans.

Notably, the maximum detachment force of our trilayer films appeared higher if compared to the one of other buccal films whose mucoadhesion was tested on porcine mucosa. As an example, Alves et al. prepared bilayer buccal films whose mucoadhesive layer included sodium carboxymethylcellulose, chitosan and methylcellulose and tested them both on mucin discs and on porcine mucosa, revealing maximum detachment forces up to 4 kPa [62].

**Figure 11:**
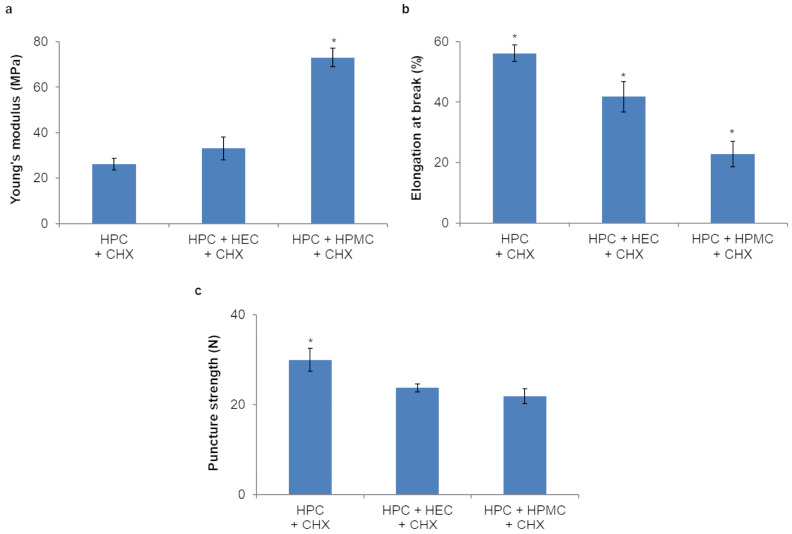
YM (**a**), EB (**b**) and puncture strength (**c**) of trilayer films (only the composition of the backing layer is indicated in the legend; values represented as mean ± SD, *n* = 3; the symbol (*) denotes statistical significance from the other compositions (*p* < 0.05)).

The studied trilayer films did not show significant differences (*p* > 0.05) in YS, but those containing HPC GF alone and HPC GF + HEC in the third layer were more elastic and elongated more at break than those containing HPC GF + HPMC (*p* < 0.05) (Figure 11a,b). Overall, the addition of release-controlling polymers to the backing layer apparently reduces the whole elasticity of the film. This evidence is confirmed by the puncture strength results (Figure 11c), which indicate a significantly higher resistance to perforation for the films not including either HEC or HPMC (*p* < 0.05). The EB of the studied films, comprised between 25 and 55%, might be considered ideal since such values indicate a good balance between flexibility and elasticity [63]; other CHX-containing buccal films described in the literature, like the CMC/microcrystalline cellulose films described by Tarawneh et al. [64], present lower EB values, which might lead to film breaking and possibly favor suitable environment for bacterial adherence [65].

The swelling index of trilayer films after 30, 60, 120 and 180 min was measured using the Petri dish method [66] in pH 6.8 PBS.

**Figure 12:**
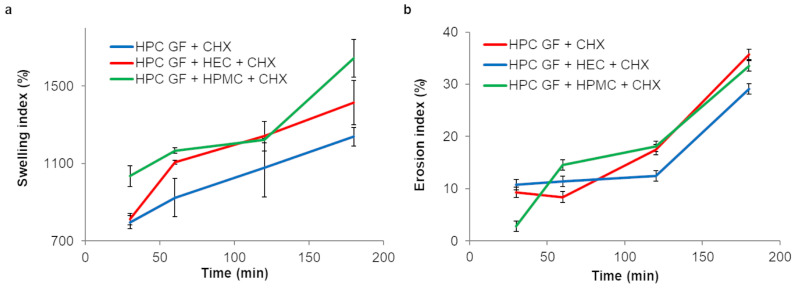
Swelling (**a**) and erosion (**b**) indexes of the prepared trilayer films tested in pH 6.8 PBS (only the composition of the backing layer is indicated in the legend; values represented as mean ± SD, *n* = 3).

The films showed a high degree of swelling (Figure 12), while still maintaining their structural integrity for an appropriate time period. The higher SI values (1644 ± 98%) after 180 min were obtained from films containing HPC GF + HPMC as the third layer, which showed also a higher final EI (33 ± 4%). On the whole, the EI was low in the case of films containing HPC G alone as the third layer.

The in vitro and in vivo residence time of trilayer films is presented in Table 3. These results are comparable to the ones of other buccal films described in the literature [56]. Interestingly, the different composition of the release control layer influences this parameter. Because of its moderate thickness and weight (75 mg), no volunteer involved in the in vivo evaluation felt the heaviness of the buccal patch at the place of attachment.

**Figure 13:**
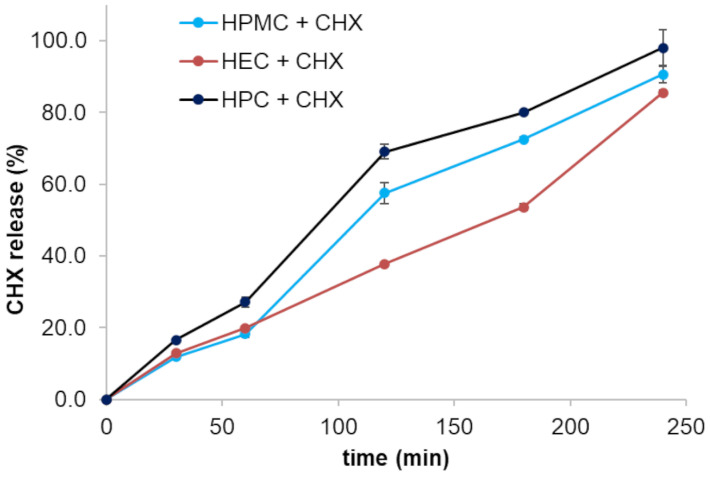
In vitro drug release profiles of the prepared trilayer films tested in simulated saliva (only the composition of the backing layer is indicated in the legend; values represented as mean ± SD, *n* = 3).

In vitro release profiles of CHX from trilayer films measured with a homemade device are shown in Figure 13. These results are comparable with the ones reported by Juliano et al. [61], relevant to films containing 5 mg CHX, the same amount loaded in the films described herein. For the multilayer film where the backing layer contained just HPC GF, the release was significantly faster, confirming the ability of HEC and HPMC to control the release rate of the loaded actives; the films of neat HPC GF released approx. 98 ± 5% of loaded CHX after 4 h, statistically more than the drug released from the HEC-containing films (85 ± 1%) and from the HPMC-containing films (91 ± 2%), suggesting that both polymers in the third layer adequately and continuously controlled the release during the film residence on the mucosa, by producing a water-swollen gel-like state that could substantially reduce the rate of penetration of the dissolution medium into the film. The slowdown effect in the drug release caused by HEC and HPMC observed in this assay might be related to some interactions with the HPC present in the backing layer, since Juliano et al. evidenced that HPMC alone was not able to control the drug release, and observed that a film composed of HPMC and glycerol led to a fast CHX burst release of 80% after only 30 min [61].

Finally, to validate the aseptic manufacturing procedure, the three formulations of trilayer films underwent a sterility test that did not show any growth of microorganisms for up to 14 days, indicating that the films thus prepared do not require terminal sterilization and could be produced as compounding formulations on a laboratory scale.

These results are evidently preliminary, as the release profile should be confirmed on human volunteers by applying the film and analyzing samples of saliva at various time points to verify whether the rate is adequate for the intended use; in fact, the film should release all its drug content within its maximum residence time. Also, the healing properties should be verified in vivo, to confirm the effectiveness of SESM on buccal epithelium regeneration.

## 4. Conclusions

The rational design of the multilayer film described in this work led us to formulate a flexible mucoadhesive film suitable for chlorhexidine digluconate delivery in the oral cavity for the treatment of oral diseases and wound-healing applications.

Preliminary preformulation studies enabled the selection of the best film-forming polymers. Biological studies on soluble eggshell membrane have successfully demonstrated its anti-inflammatory and wound-healing properties and its potential role in enhancing the therapeutic efficacy of the film.

The mucoadhesive layer containing HPC GF and SESM showed good mucoadhesion and an absence of interference with blood clotting. The supportive layer containing HPC GF with gelatin enhanced the mechanical properties of the multilayer films for their practical detachment from the substrate and handling purposes. The backing/drug delivery layer containing HPC GF, when mixed with HEC or, to a lesser extent, HPMC, allowed for the controlled release of chlorhexidine digluconate, which could reduce the risk of bacterial infection at the wound site, but, as it was incorporated in the outer layer, might exert minimal or no interference with the healing properties of SESM.

The good swelling index and convenient in vitro and in vivo residence time of the trilayer films ensured prolonged drug release. Furthermore, ex vivo studies on porcine mucosa and in vivo evaluation on volunteers provided information on the possible adhesion of our final formulation to oral lesions or dental complications; therefore, an optimization study on these multilayer films for effective therapeutic uses is currently underway.

The ease of preparation, the adhesion and release control properties and the protective function suggest potential applications of our multilayer films for wound healing in the oral cavity, for the treatment of aphthous ulcers and lesions related to periodontal disease.

## Figures and Tables

**Table 1:** Measured parameters for the films obtained from the polymers selected at the first stage of preformulation study. YS = yield strength, YM = Young’s modulus, EB = elongation at break.

	Maximum Detachment Force (MPa)	Work of Adhesion (mJ)	YS (MPa)	YM (MPa)	EB (%)
HEC G	0.059 ± 0.002	3.0 ± 0.1	19 ± 3	131 ± 66	17 ± 7
HEC L	0.062 ± 0.006	3.3 ± 0.6	9 ± 2	186 ± 65	5 ± 1
HPC GF	0.068 ± 0.004	4.0 ± 0.1	16 ± 2	11 ± 1	154 ± 3
HPMC E15	0.064 ± 0.004	4.5 ± 0.3	75 ± 3	222 ± 34	34 ± 6
HPMC K100LV	0.062 ± 0.005	4.3 ± 0.6	20 ± 1	254 ± 45	8 ± 1
HPMC K750	0.059 ± 0.002	4.0 ± 0.4	107 ± 14	341 ± 69	32 ± 2
PVA 18-88	0.069 ± 0.002	4.2 ± 0.5	8 ± 2	94 ± 58	9 ± 3
PVA SRP 80	0.069 ± 0.004	4.4 ± 0.5	17 ± 3	190 ± 62	9 ± 1

**Table 2:** Composition and physical characteristics of trilayer films.

Trilayer Film Composition			Weight (mg) *	Thickness (mm) *
Mucoadhesive Layer	Supporting Layer	Backing Layer		
HPC GF + SESM 70/30 (SESM 7.5 mg/film)	HPC GF + GEL 70/30	HPC GF + CHX 83.3/16.7 (CHX 5 mg/film)	76 ± 1	0.16 ± 0.01
		HPC GF + HEC G + CHX 41.65/41.65/16.7 (CHX 5 mg/film)	75 ± 1	0.17 ± 0.01
		HPC G + HPMC K750 + CHX 41.65/41.65/16.7 (CHX 5 mg/film)	75 ± 1	0.16 ± 0.01

* values represented as mean ± SD (*n* = 5).

**Table 3:** In vitro and in vivo residence time of the trilayer films—values represented as mean ± SD, *n* = 3; the symbol (*) denotes statistical significance from the other compositions (*p* < 0.05).

Formulation	In Vitro Residence Time (min)	In Vivo Residence Time (min) ^a^
HPC GF + SESM/HPC GF + GEL/HPC GF (+CHX)	174 ± 11 *	104 ± 10 *
HPC GF + SESM/HPC GF + GEL/HPC GF + HEC (+CHX)	274 ± 9 *	222 ± 19 *
HPC GF + SESM/HPC GF + GEL/HPC GF + HPMC (+CHX)	226 ± 6	145 ± 7

^a^ the films tested for in vitro residence time included CHX in the backing layer; the films tested for in vivo residence time did not include CHX.

## Data Availability

Data are contained within the article or Supplementary Material.

## Supplementary Materials

The following supporting information can be downloaded at: www.mdpi.com/article/10.3390/pharmaceutics16101342/s1, Table S1: List of polymers tested and relevant film properties.
