# Supplementary material for: Development of a Multilayer Film Including the Soluble Eggshell Membrane Fraction for the Treatment of Oral Mucosa Lesions
Source: Pharmaceutics. 2024 Oct 19;16(10):1342. doi: 10.3390/pharmaceutics16101342 (PMC11511083; doi:10.3390/pharmaceutics16101342)
Supplement: Supplementary file 1 [file pharmaceutics-16-01342-s001.zip › pharmaceutics-3229252-supplementary.pdf]

pharmaceutics

Supplementary Materials: Development of a Multilayer Film Including the Soluble Eggshell Membrane Fraction for the Treatment of Oral Mucosa Lesions

Karthik Neduri, Giorgia Ailuno, Guendalina Zuccari, Anna Maria Bassi, Stefania Vernazza, Anna Maria Schito, Gabriele Caviglioli and Sara Baldassari

Table S1. List of polymers tested and relevant film properties; the polymers selected for further evaluation are written in bold.

Polymer	Flexibility / rigidity	Detachability	Appearance
**HEC L**	**Flexible**	**Detachable**	**Translucent**
**HEC G**	**Flexible**	**Detachable**	**Transparent**
HEC HHX	Too viscous to cast	N/A	N/A
**HPC G**	**Flexible**	**Detachable**	**Transparent**
HPC M	Too viscous to cast	N/A	N/A
HPMC E3	Flexible	Detachable	Transparent
**HPMC E15**	**Flexible**	**Detachable**	**Transparent**
HPMC K15M	Too viscous to cast	N/A	N/A
**HPMC K100LV**	**Flexible**	**Detachable**	**Translucent**
HPMC K250	Flexible	Detachable	Translucent
**HPMC K750**	**Flexible**	**Detachable**	**Translucent**
CMC 7LP	Rigid	Detachable	Transparent
CMC 7M8SF	Rigid	Detachable	Transparent
MC A4M	Partially flexible to rigid	Detachable	Transparent
MC A15 LV	Partially flexible to rigid	Detachable	Transparent
**PVA 18-88**	**Flexible**	**Detachable**	**Translucent**
**PVA SRP 80**	**Flexible**	**Detachable**	**Transparent**
PVP K30	Brittle	Undetachable	Transparent
PVP K90	Flexible	Detachable	Transparent
PVP-VA64	Brittle	Undetachable	N/A
PEO WSR N-80	Flexible	Detachable	Translucent
PEO 1000 kDa	Flexible	Detachable	Translucent
PEO 2000 kDa	Flexible but shrunken	Detachable	Translucent
Pectin HM	Rigid	Detachable	Translucent
Carbopol 971	Too viscous to cast	N/A	N/A
Poloxamer P407	Brittle	Detachable	Translucent
Sodium alginate	Rigid	Detachable	Translucent
HPSP	Flexible	Detachable	Translucent
